# Ultrasound-Assisted Deep Eutectic Solvents Extraction of Polysaccharides From *Morchella importuna*: Optimization, Physicochemical Properties, and Bioactivities

**DOI:** 10.3389/fnut.2022.912014

**Published:** 2022-06-09

**Authors:** Xu Pan, Lijing Xu, Junlong Meng, Mingchang Chang, Yanfen Cheng, Xueran Geng, Dongdong Guo, Rongzhu Liu

**Affiliations:** ^1^College of Food Science and Engineering, Shanxi Agricultural University, Jinzhong, China; ^2^Shanxi Key Laboratory of Edible Fungi for Loess Plateau, Taigu, China; ^3^Shanxi Engineering Research Center of Edible Fungi, Taigu, China

**Keywords:** *Morchella importuna*, polysaccharides, deep eutectic solvent, ultrasound-assisted extraction, antioxidant activity, hypoglycemic activity

## Abstract

In this study, a high-efficiency and non-pollution extraction procedure, ultrasound-assisted technique with deep eutectic solvents (DESs), was applied for extraction of polysaccharides from *Morchella importuna* (MIP-D). The results exhibited that the system of DES was: mole ratio between choline chloride and oxalic acid of 2:1, water content of 90% (v/v), and the optimal extraction parameters were as follows: extraction time of 31.2 min, extraction temperature of 62.1^°^C, and the liquid–solid ratio of 32.5:1 (v/w). Under these extraction parameters, the extraction yield of MIP-D was 4.5 times higher than hot water extraction (HWE) method and had higher carbohydrate (85.27%) and sulfate contents (34.16%). Moreover, high-performance liquid chromatography (HPLC) and Fourier-transform IR (FTIR) spectrum analysis indicated that MIP-D was comprised of glucosamine, galactose, glucose, and mannose, with molar ratios of 0.39:1.88:3.82:3.91, which contained the pyranose ring skeleton. High-performance gel permeation chromatography (HPGPC) analysis revealed that MIP-D showed three fractions with molecular weights of 2.6 × 10^6^, 7.3 × 10^4^, and 3.7 × 10^3^ Da, which were lower than those of polysaccharides extracted by HWE. *In-vitro* tests proved that MIP-D possessed excellent antioxidant and inhibited α-amylase and α-glucosidase inhibitory activities. Therefore, DESs (choline chloride-oxalic acid) as a high-efficiency and non-pollution solvent alternative can be applied to the separation of bioactive polysaccharides from *Morchella importuna* (*M. importuna*).

## Introduction

Polysaccharide, comprising of more than ten monosaccharides, is one of the four basic substances that make up life, found in animal cell membranes and cell walls of plants and microorganisms (1). Previous studies have also demonstrated that polysaccharide has the effects of antitumor, antifatigue, postponing aging, reducing blood lipid, hypoglycemic activity, and so on (2, 3). Moreover, traditional separation methods of polysaccharides from natural materials have been associated with longer extraction time, lower efficiency, and environmentally disastrous (4). Thus, efficient and green extraction methods of polysaccharides are required to overcome these problems.

The most effective methods for separating polysaccharides are chemical treatment. Chemical methods generally use strong alkali and acid to remove impurities, respectively, so they are not environmentally friendly. Compared with chemical methods, biological methods using enzymes are more environmentally friendly, but these methods not only have low extraction efficiency, but also are not conducive to large-scale industrial production (5). Recently, deep eutectic solvents (DESs) have been found to be composed of hydrogen bond donors (HBDs) and hydrogen bond acceptors (HBAs) in a certain proportion (6). They can provide or accept external electrons or protons to form hydrogen bonds (4). Therefore, they can dissolve a variety of substances, such as polysaccharides and salts (7). In addition, deep eutectic solvents (DESs) not only have good properties of ionic liquids, such as excellent solubility, chemical stability, and thermal stability (8), but also have simple preparation, low cost, and no pollution (9). Currently, some studies have shown that DESs can be used as alternative solvents to extract polysaccharides from natural samples.

*Morchella importuna* (*M. importuna*), belonging to the Ascomycota, is widely distributed in Europe, Asia, and America and known as one of the rare and precious edible fungi in the world (10). Previous studies have also demonstrated that polysaccharides are one of the main bioactive components in *M. importuna* (11). However, extraction of polysaccharides from *M. importuna* has been rarely reported. Especially, the effect of choline chloride-oxalic acid (ChCl-OA) as an extraction medium on the physicochemical properties and bioactivities of *M. importuna* polysaccharides (MIPs) has not been studied.

Therefore, a high-efficiency and non-pollution extraction procedure was developed for the extraction of polysaccharides from *Morchella importuna* by ultrasound-assisted extraction (UAE). The optimum extraction conditions were optimized with the response surface methodology (RSM) employing Box–Behnken design (BBD). Subsequently, the physical–chemical characteristics of MIP-D were investigated by high-performance liquid chromatography (HPLC), high-performance gel permeation chromatography (HPGPC), and Fourier-transform IR (FTIR) and the biological activities of MIP-D were evaluated based on its antioxidant and hypoglycemic abilities.

## Materials and Methods

### Materials and Chemical Reagents

Standard monosaccharides were purchased from Sigma Chemical Corporation (United States). Reagents containing choline chloride, oxalic acid dihydrate, citric acid, glycerol, 1,4-butanediol, acetamide, urea, anhydrous ethanol, citric acid, α-amylase, α-glucosidase, 1,1-diphenyl-2-picrylhydrazyl (DPPH), 2,2′-Azinobis-(3-ethylbenzthiazoline-6-sulphonate) (ABTS), and OH assay kit were purchased from Solarbio Science (Beijing, China). Other chemicals used throughout the experiment were analytical grade.

### Extraction Methods

#### Deep Eutectic Solvent Preparation

The preparation of DESs was conducted as previously described (12). Six different types of DES were prepared by the heating method and used as solvent for ultrasonic-assisted extraction of MIPs instead of water. The types of DESs are shown in Supplementary Table 1. DESs were produced by mixing HBDs and HBAs in water bath at 80^°^C according to a certain molar ratio until they were completely dissolved and a colorless and transparent thick liquid was formed at room temperature.

#### Preparation of *M. importuna* Polysaccharides

The *M. importuna* was dried to constant weight and crushed with a grinder (Zhejiang Hongjingtian Corporation Ltd.) and the powder was obtained through the 50-size mesh. The extraction process was performed in an ultrasonic cleaner (KQ5200DE type, 40 kHz, 10 L, 600 W, Kunshan Ultrasonic Instrument Corporation Ltd.).

The powder was mixed with DES that had been prepared and the mixture was incubated at a constant temperature, followed by the collection of the supernatant *via* centrifugation under 5,000 *g* for 10 min (Allegra 64R Centrifuge, Beckman Coulter Incorporation). Then, the protein in the supernatant was removed by the potassium ferrocyanide-zinc acetate method and dialyzed with distilled water for 24 h. The quadruple volume of dehydrated ethanol was added in the resulting solution, which was then placed at 4^°^C overnight to complete precipitation and then collected the precipitation, dissolved it with an appropriate amount of water and freeze-dried to obtain the crude polysaccharide from *M. importuna*.

In order to evaluate the UAE of MIPs, the polysaccharide from *M. importuna* by hot water method (MIP-W) was studied. The *M. importuna* powder (1 g) was added to 30 ml of distilled water and incubated at 80^°^C for 2 h. After repeated extraction for three times, the supernatant was combined, dialyzed, precipitated, and dried according to the above method.

### Optimization of Ultrasound-Assisted Extraction

Effects of DES-1 mole ratio (1.5:1, 2:1, 2.5:1, 3:1, and 3.5:1), water content of DES-1 (10, 30, 50, 70, 90, and 100%, w/w), extraction time (10, 20, 30, 40, and 50 min), extraction temperature (40, 50, 60, 70, and 80°C), and liquid–solid ratio (10, 20, 30, 40, and 50 v/w) on the extraction efficiency of MIPs were studied by single factor test. Under the conditions of other factors remaining unchanged, each experimental factor was optimized. Finally, the extraction time, extraction temperature, and liquid–solid ratio were determined as the main factors. Based on the results from the above experiment, the response surface methodology (RSM) employing Box–Behnken design (BBD) was used to optimize the three main parameters and the extraction efficiency of MIPs was regarded as the response. The experimental designs of the code levels of the three influencing factors were nominated as (−1, 0, and +1) and actual experimental values of each variable are shown in Supplementary Table 2. Each experiment was carried out three times. The experimental data obtained from BBD were analyzed by regression analysis. Under the optimal parameters, the extraction experiment was repeated for three times to verify the accuracy of RSM. The polysaccharide from *M. importuna* (MIP-D) was extracted under optimized conditions to determine their structural characterizations and bioactivities.

### Structural Characterizations and Bioactivities of *Morchella importuna*

#### Chemical Composition Determination

The extraction efficiency of MIPs (%) was determined as follows: the extraction efficiency of MIPs (%) = [Weight of dried MIPs (g) × Total carbohydrate content of MIPs (%)/Weight of dried *M. importuna* (g)]. The content of carbohydrate, protein, uronic acid, and polyphenol in MIPs was measured by the phenol-sulfuric acid method with glucose as a standard, Bradford method with bovine serum albumin (BSA) as a standard, sulfuric acid-carbazole method with galacturonic acid as a standard, and the Folin–Ciocalteu method with pyrogallic acid as a standard based on our previous study (13, 14). The content of sulfate radical was determined by ion chromatography method with potassium sulfate as a standard according to the reported method (15).

#### Monosaccharide Composition Determination

The constituent monosaccharides of MIPs were measured by HPLC analysis based on our previous study, which is slightly modified (16). The eluents were NaOH (15 mM) flowing at 0.3 ml/min and 5 μl sample (0.1 mg/ml) was injected at 30^°^C.

#### Molecular Weight Determination

The weight-average molecular weight (Mw) of the MIPs was measured by HPGPC analysis on the Agilent 1260 Series HPLC System, equipped with series-connected Ultrahydrogel™ 2000 and Ultrahydrogel™ 500 columns and refractive index detector (RID) (17). The column was calibrated by standard dextrans (T-3, T-10, T-40, T-70, and T-110 series). The eluent was 0.1 mol/l NaNO_3_, which was injected at 0.5 ml/min and 20 μl sample (5.0 mg/ml) was injected at 35^°^C.

#### Ultraviolet Spectrum Analysis

Ultraviolet spectrophotometer (LabTech, UV9100A, United States) was employed to determine the protein content in range of 200–400 nm.

#### Fourier-Transform IR Spectroscopy

The MIPs were mixed and grounded with KBr at a weight ratio of 1:100, followed by pressing into tablets. Spectrometer (Tensor 27, Bruker, Germany) was employed to analyze the FTIR spectra of MIPs in the wavenumber region of 4,000–5,000 cm^–1^.

### *In-vitro* Antioxidant Activities Assays

The DPPH, ABTS, and OH radical scavenging abilities of MIP-D were measured strictly based on the methods of DPPH, ABTS, and OH kit, respectively.

### *In-vitro* α-Amylase and α-Glucosidase Inhibitory Activities

The α-amylase and α-glucosidase inhibitory activities of MIPs were measured according to the reported method (18).

## Results and Discussion

### Optimization of Ultrasound-Assisted Extraction

#### Effect of Deep Eutectic Solvent System

**FIGURE 1:**
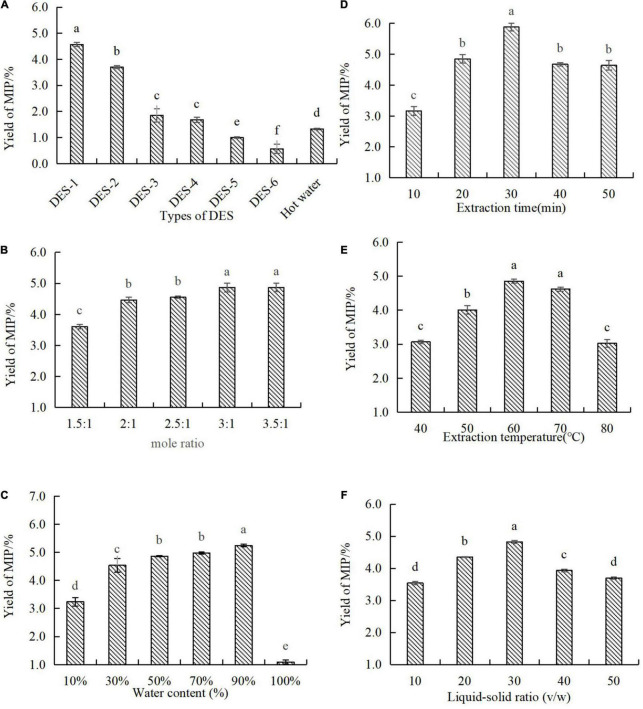
Effect of different extraction parameters in the extraction yield of MIP-D. **(A)** Types of DESs, **(B)** mole ratio, **(C)** water content, **(D)** extraction time, **(E)** extraction temperature, and **(F)** ratio of liquid–solid. Data represent means ± *SD* (*n* = 3). Significant (*p* < 0.05) differences are shown by data bearing different letters (a–d).

In the process of extracting target bioactive substances from the sample, the physicochemical properties of the extraction solvent were essential conditions for this study, which might be due to the permeability of the extractant in the sample matrix that can be determined directly by them (4). According to different hydrogen bond donors, six different types of DES were synthesized in order to select a suitable DES for the extraction of MIPs (19). As shown in Figure 1A, the extraction efficiency varied with different types of DES. By comparing the hot water extraction (HWE), DES-1 and DES-2 increased the extraction efficiency of MIPs by 3.4 and 2.8 times, respectively. There was no significant difference between DES-3 and DES-4 in the extraction yields of MIPs. Effects of DES-5 and DES-6 on the extraction yields of MIPs were significantly lower than those obtained by hot water. DES-1 exhibited higher extraction efficiency than others, which might be attributed to its stronger electrostatic interaction between DES-1 and MIPs than those of other DESs (20). Furthermore, oxalic acid and choline chloride are non-toxic substances and widely found in natural foods (21). Therefore, the DES system constructed by choline chloride-oxalic acid was considered for the follow-up experiments.

#### Effect of Mole Ratio of Deep Eutectic Solvent

As reported, the hydrogen bond interaction of DES was determined by the molar ratio of HBD and HBA in the deep eutectic solvent, which affects the viscosity and surface tension of DES. The physical properties of DES would further affect the extraction efficiency of polysaccharides (4). It was important to note that DES was crystallized when the molar ratio was more than 3.5:1, so increasing the content of choline chloride was not attempted. As shown in Figure 1B, we can see that the extraction efficiency of MIPs was significantly affected by the molar ratio of DES-1. Moreover, the extraction efficiency increased with the increasing ratio of choline chloride in DES-1, which might be due to choline chloride improved the viscosity and surface tension of DES-1, thus increasing the permeability of DES-1 in *M. importuna* (4). However, a large number of crystals were produced in the process of alcohol precipitation with a molar ratio of 2.5–3.5:1 in DES-1, which made the subsequent extraction procedure into trouble. Due to this, the mole ratio of ChCl-OA in DES-1 used in the subsequent experiments was 2:1.

#### Effect of Water Content of Deep Eutectic Solvent

Previous studies have also revealed that the high viscosity and weak mobility of DES would reduce the extraction efficiency of polysaccharides. However, the viscosity and polarity of DES were changed by the water content of DES system (22). Thus, it is essential to choose a suitable water content to improve the mass transfer rate and the processing efficiency. As shown in Figure 1C, extraction efficiency varied considerably dependent on DES water content. The extraction efficiency increased significantly with the increasing of the water content of DES-1 from 10 to 90%. This phenomenon might be attributed to the increase of water content in DES-1 leaded to a more suitable viscosity and polarity of DES-1 to improve interactions between polysaccharides and DES-1, which obtained a high extraction yield (4). However, the further increase of water content (100%) resulted in a significant decrease in extraction efficiency. In addition, DES-1 with the water content of 90% was not only easier to produce because of its low viscosity and high fluidity, but also had a lower cost. Therefore, DES-1 with the water content of 90% was considered for the subsequent experiments.

#### Effect of Extraction Time

Previous studies had shown that the extraction efficiency was generally proportional to the extraction time (23). As shown in Figure 1D, the extraction efficiency of MIPs was affected by prolonging the extraction time. With the extension of extraction time, the extraction efficiency of MIP increased significantly from 3.16 to 5.88%, which might be due to the prolonging of extraction time that can destroy the cellular structure, increase the permeability, and can be conducive to polysaccharides extraction (23). However, when the extraction time increased from 30 to 50 min, the extraction efficiency of MIP dropped from 5.88 to 4.63%. This phenomenon might be related to the hydrolysis of MIPs at high temperature for a long time (24). Hence, the extraction time was selected in 30 min for the subsequent experiments.

#### Effect of Extraction Temperature

As reported, the extraction temperature is one of the vital factors influencing the extraction yield of polysaccharides (25). As shown in Figure 1E, the extraction efficiency of MIPs increased with the increase of extraction temperature from 40 to 60^°^C and reached the maximum polysaccharides yield (4.85%) at 60^°^C. This phenomenon might be due to the high temperature that may not only decrease the viscosity of DES-1 and increase the diffusion of DES-1, but also accelerate the mass transport of polysaccharides (19), which makes polysaccharides dissolve more in solvent and improve extraction yield. However, the higher temperature may decrease physical adsorption between the DES and MIPs (21), which will cause the target compounds to be separated from the DES hydrogen bond network. Thus, the extraction efficiency of MIPs decreased from 4.85 to 3.03%. In conclusion, the extraction temperature was selected at 60°C for the subsequent experiments.

### Effect of Liquid–Solid Ratio

Study indicated that the liquid–solid ratio could affect the extraction efficiency of polysaccharides (20). As shown in Figure 1F, the effect of liquid–solid ratios on the extraction efficiency of MIPs was measured in this experiment. The extraction efficiency of MIPs increased with the increase of liquid–solid ratio from 10:1 to 50:1 and reached the maximum (4.82%) at 30:1 (v/w). However, a further increase of the liquid–solid ratio resulted in lower extraction efficiency. This phenomenon might be due to a small amount of DES-1 that could approach the extraction equilibrium, while a large amount of DES could increase the leaching of other compounds, resulting in the decrease of yield of MIPs. Moreover, the increase of liquid–solid ratio not only enhanced the difficulty of extracting polysaccharides, but also caused wastage of DES-1. Thus, 30:1 (v/w) was considered as the optimal liquid–solid ratio in this experiment.

In a word, the optimal system of DES was: a mole ratio between ChCl and OA of 2:1 and water content of 90% (v/v). Moreover, the preliminary optimization of experimental factors was: extraction time of 30 min, extraction temperature of 60^°^C, and liquid–solid ratio of 30:1 (v/w).

### Optimization of Ultrasound-Assisted Extraction

#### Statistical Analysis

Based on the results of single-factor experiment, RSM was applied to study the influencing factors of UAE employing Design-Expert software (version 10.0.7). The data of 17 runs with three factors and three levels for optimizing the mutual effects of three parameters are shown in Supplementary Table 3. The data were analyzed by multiple regression analysis and the predicted MIPs yield was calculated using following equation:

$$Y=5.84+0.19\,A+0.37\,B+0.14\,C-0.23\,A\,B+0.005976\,A\,C-0.097\,B\,C-0.60\,A^{2}-0.76\,B^{2}-0.24\,C^{2}$$

The results of the statistical summary are shown in Table 1. The F-value of the model was 52.33 (*P* < 0.0001), exhibiting that the model was significant (26). Additionally, the values of the determination coefficient (*R*^2^) and adjusted determination coefficient (*R*^2^_adj_) were 0.9854 and 0.9665, respectively, which confirmed the highly goodness-of-fit of the model (27). Meanwhile, the value of the coefficient of variation (CV) was 2.28, which demonstrated the excellent reliability of the data (28). The *p*-values of 0.1793 and the F-value of 2.72 for lack of fit indicated the validity of the model. The linear coefficients (A, B, and C), cross-product coefficient (AB), and the quadratic term coefficients (A^2^, B^2^, and C^2^) were all significant (*p* < 0.05), whereas cross-product coefficients (AC and BC) were not significant. We concluded that the order of three factors as follows: B (*P* < 0.0001) > A (*P* = 0.0026) > C (*P* = 0.0128).

**TABLE 1:** Chemical composition and sugar composition of MIPs.

Sample	Carbohydrate/%	Protein/%	Sulfate content/%	Polyphenols/%
MIP-D	85.27 ± 1.35	2.57 ± 0.06	34.16 ± 1.61	4.49 ± 0.39
MIP-W	57.89 ± 2.63	3.85 ± 0.03	24.43 ± 1.15	10.60 ± 0.35

**Sugar composition (molar ratio)**				

	**GlcN**	**Gal**	**Glc**	**Man**

MIP-D	0.39	1.88	3.82	3.91
MIP-W	2.71	1.06	5.59	3.01

*Each value represents the mean ± SD (n = 3).*

#### Response Surface Analysis

**FIGURE 2:**
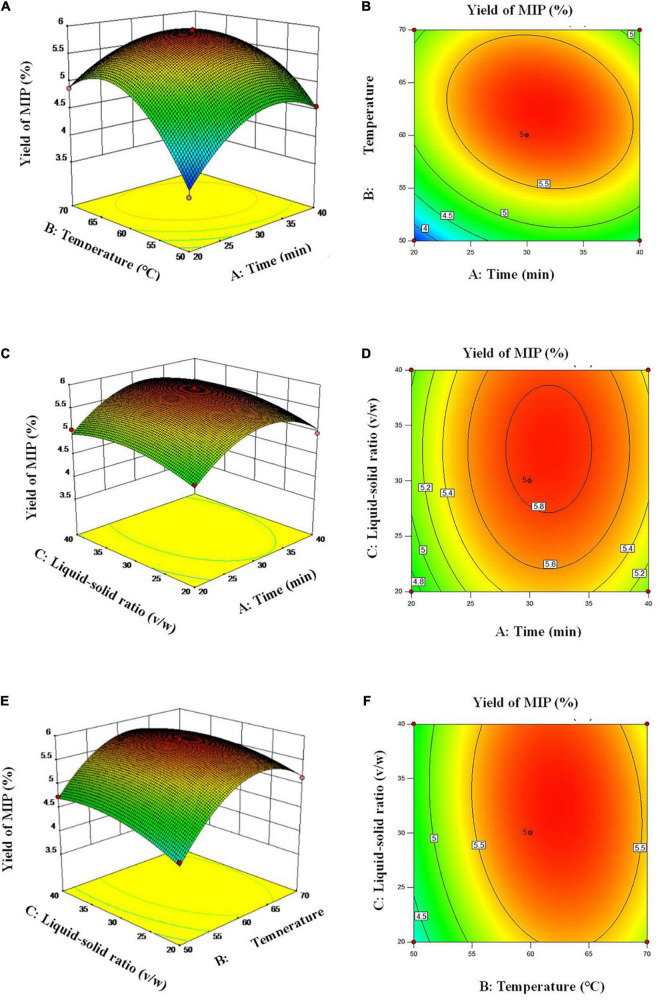
Response surface plots **(A,C,E)** and response contour plots **(B,D,F)** showing the extraction time, extraction temperature and Liquid-solid ratio on the extraction yield of MIP.

The effect of the interaction of three factors on the extraction efficiency of MIPs could be visualized by the three-dimensional (3D) response surface (3D plots) and two-dimensional (2D) contour plots (2D plots). The mutual influences of various factors on the extraction efficiency of MIPs were reflected by the 3D plots and the significance of it was displayed by the 2D plots (26). The 3D plots in Figures 2A,C,E revealed that with the increase of extraction time (A), extraction temperature (B), and liquid–solid ratio (C), the extraction efficiency of polysaccharides increased first and then decreased. The 2D of Figure 2B showed that the interaction between A and extraction temperature B is significant. This observation agreed with the results of the *p*-value of cross-product coefficient (AB). Figures 2D,F illustrate that the interaction between AC and BC was insignificant.

#### Response Surface Results Optimization

By using Design-Expert software (version 10.0.7), the optimal conditions of three parameters were obtained and proposed as a practical response variable for the extraction: extraction time (A) of 31.2 min, extraction temperature (B) of 62.1^°^C, liquid–solid ratio (C) of 32.5:1, and the maximum estimated extraction values of 5.91%. Under the optimal parameters, the extraction experiment was repeated for three times to verify the accuracy of RSM and the extraction efficiency of MIPs was 5.93 ± 0.07%. The results confirmed that the experimental values of MIPs extraction were in good agreement with the predictive values of MIPs extraction. Compared with the HWE and UAE with water instead of DES, the UAE exhibited a higher extraction efficiency (4.5 and 5.4 times higher, respectively). Therefore, the response model was suitable for UAE procedure.

#### Chemical Composition, Monosaccharide Composition, and Molecular Weight Distribution of MIPs

The chemical compositions of MIPs are shown in Table 2. The carbohydrate, protein, sulfate, and polyphenol contents of MIP-D were measured to be 85.27, 2.57, 34.16, and 4.49%, respectively. The results obtained showed that MIP-D was an acidic heteropolysaccharide containing sulfate, which might exhibit excellent biological activity (29). Hu et al. also found that *Acanthopanax leucorrhizus* polysaccharide (ALP) shows better antioxidant activities after sulfated modification (30). After 24 h of dialysis and impurity removal, 4.49% of phenolic compounds were still detected in MIP-D, which indicated that natural polyphenolic-polysaccharide conjugates might exist in MIP-D. Liu et al. suggested that the conjugation of phenolic compounds can improve the antioxidant and enzyme inhibition effects of polysaccharides (31). Moreover, uronic acid was not detected in the chemical composition of MIP-D, which was agreed with the results of the previous study (1). To further evaluate the physicochemical properties of polysaccharides extracted by UAE, MIP-W was studied. Less carbohydrate (57.89%) and sulfate (24.43%) and more protein (3.85%) and polyphenol (10.60%) were found in MIP-W, which might be related to extraction method and solvent.

**TABLE 2:** ANOVA of the experimental results of the Box–Behnken design (BBD).

Source	Sum of squares	df	Mean square	*F*-value	*p*-value
Model	6.3516	9	0.7057	52.33	< 0.0001**
A	0.2819	1	0.2819	20.90	0.0026**
B	1.0800	1	1.0800	80.08	< 0.0001**
C	0.1483	1	0.1483	10.99	0.0128*
AB	0.2167	1	0.2167	16.07	0.0051**
AC	0.0001	1	0.0001	0.01	0.9210
BC	0.0375	1	0.0375	2.78	0.1395
A^2^	1.5003	1	1.5003	111.25	< 0.0001**
B^2^	2.4579	1	2.4579	182.26	< 0.0001**
C^2^	0.2375	1	0.2375	17.61	0.0041**
Residual	0.0944	7	0.0135		
Lack of fit	0.0633	3	0.0211	2.72	0.1793
Pure error	0.0311	4	0.0078		
Cor total	6.4460	16			

CV%, 2.28; R^2^, 0.9854; R^2^_ad_, 0.9665. *p < 0.05, **p < 0.01.

**FIGURE 3:**
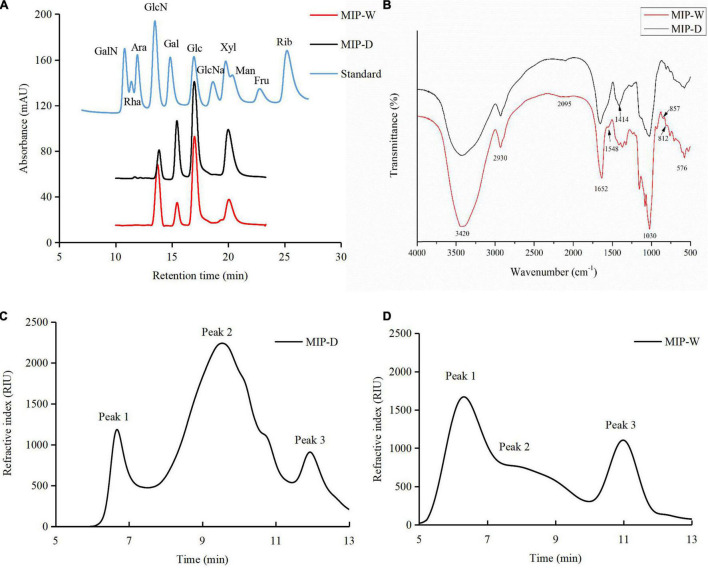
**(A)** High performance liquid chromatography profiles of MIPs and monosaccharide standard; **(B)** fourier-transform IR (FTIR) spectra of MIPs over the frequency range of 4,000–5,000 cm^–1^; high-performance gel permeation chromatography profiles of MIP-D **(C)** and MIP-W **(D)**.

As shown in Table 2 and Figure 3A, MIP-D and MIP-W were composed of glucosamine (GlcN), galactose (Gal), glucose (Glc), and mannose (Man) with molar ratios of 0.39:1.88:3.82:3.91 and 2.71:1.06:5.59:3.01, respectively, according to elution time of monosaccharides standards. Moreover, uronic acid was not measured in the monosaccharide composition of MIPs, which was consistent with the results of chemical composition. Results suggested that UAE did not affect the monosaccharide composition of MIPs, but affected their molar ratios.

As shown in Figures 3C,D, MIP-D and MIP-W showed three peaks. The Mw values of MIP-D were measured to be 2.6 × 10^6^, 7.3 × 10^4^, and 3.7 × 10^3^ Da and the Mw values of MIP-W were measured to be 4.1 × 10^6^, 1.0 × 10^6^, and 3.5 × 10^3^ Da, respectively, according to the standard curve (*y* = −0.5412x + 10.086, *R*^2^ = 0.994). Furthermore, the Mw of MIP-D was lower than MIP-W, which might be due to some high molecular weight fractions were ultrasonically degraded (32). Similar reports were also observed in previous studies (33). Therefore, these results indicated that UAE extraction method might extract lower molecular weight fractions of polysaccharides than the traditional HE extraction.

### Ultraviolet and Fourier-Transform IR Spectroscopy

As shown in Supplementary Figure 1, MIP-D and MIP-W had no absorption peak at 260 and 280 nm, which indicated the absence of proteins and nucleic acids in MIPs. However, the protein contents were 2.57 and 3.85% in the chemical compositions of MIP-D and MIP-W, which might be related to the protein of MIPs being below the minimum detection of UV spectrophotometer.

The FTIR spectra were also applied for the determination of structural features of MIPs. As shown in Figure 3B, the absorption peaks at approximately 3,420 and 2,930 cm^–1^ were caused by O-H and C-H stretching vibration, respectively. The peak at 1,652 cm^–1^ was related to the C = O bending vibration (34). However, MIP-W had the absorption peak at 1,548 cm^–1^, whereas MIP-W did not, indicating that MIP-W contained more protein, consistent with prior results (Table 2). Furthermore, the peak at 1,414 cm^–1^ was caused by the C = C bending vibration (34) and the weak peak at 1,253 cm^–1^ was assigned to the asymmetry stretching vibration of S = O, which indicated that MIPs contained sulfate radical (29). The peak at 1,030 cm^–1^ represented the C-O-C asymmetric stretching vibration, indicating the presence of the pyranose ring skeleton in MIPs (14). Finally, the weak peaks at about 850 and 812 cm^–1^, which might be attributed to the existence of α-type glycosidic linkages (29). Our results suggested that MIPs had the same FTIR spectra characteristic.

### *In-vitro* Antioxidant Activity of the *Morchella importuna*

The DPPH is widely applied to measure the radical-scavenging ability of different bioactive substances separated from food products because of its stability (35). The ABTS is one of the vital radicals for determining the antioxidant ability of various bioactive substances from food products (36). Hydroxyl radical is one of the most toxic free radicals because it is associated with oxidative damage biomolecules in cells, such as alcohols, sugars, nucleic acids, and proteins, which can lead to tissue damage and even cell death (37). Therefore, to find bioactive substances that can remove OH radicals in nature, it is also very important.

**FIGURE 4:**
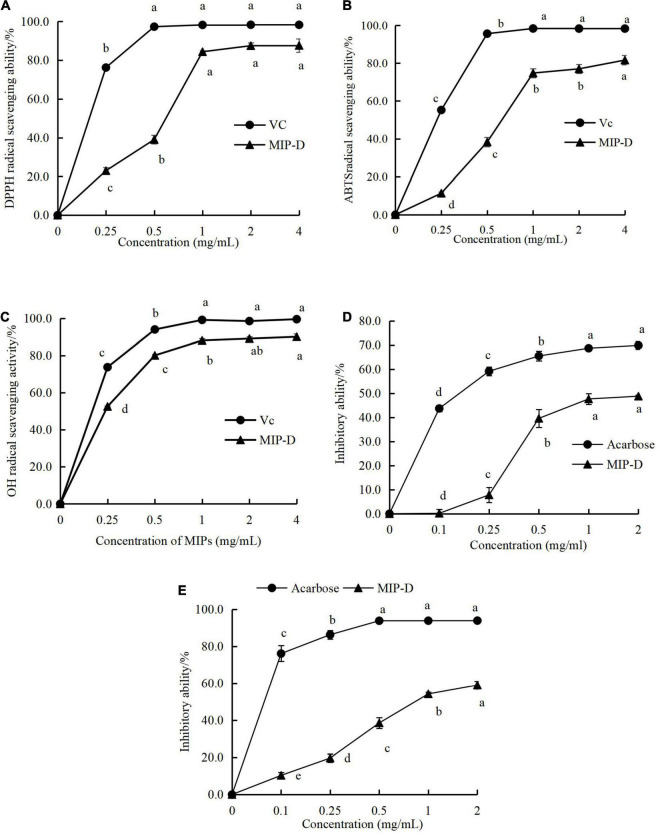
Effect of MIP-D on antioxidant and hypoglycemic activity *in vitro*. **(A)** DPPH radical scavenging activity of MIP-D. **(B)** ABTS radical scavenging activity of MIP-D. **(C)** OH radical scavenging activity of MIP-D. **(D)** The inhibitory effects of MIP-D on α-glucosidase and **(E)** the inhibitory effects of MIP-D on α-amylase. Each value represents the mean ± *SD* (*n* = 3). Significant (*p* < 0.05) differences are shown by data bearing different letters (a–d).

The antioxidant activities of MIP-D are shown in Figures 4A–C. The DPPH, ABTS, and OH radical scavenging activities of MIP-D increased with increasing concentrations in the measurement range (0–4 mg/ml) and the highest radical scavenging activities were observed at concentrations of 4 mg/ml, which were 87.64, 81.61, and 89.39%, respectively. Furthermore, the IC_50_ values of the DPPH, ABTS, and OH radical scavenging activities of MIP-D were estimated to be approximately 0.55, 0.75, and 0.15 mg/ml, respectively, which were weaker than Vc (Supplementary Table 4).

In summary, the results indicated that the excellent antioxidant activities *in vitro* were observed in MIP-D and this phenomenon might be attributed to the content of sulfate radical and phenolic compounds that bonded with polysaccharides in MIP-D. Studies suggested that sulfated polysaccharides enhance its antioxidant activity by improving the hydrogen or electron donor capacity of its derivatives and the conjugation of phenolic compounds can improve the antioxidant nature of polysaccharides (30, 31). However, the antioxidant mechanism and the structure–activity relationship of MIP-D are not clear and need further study.

### *In-vitro* α-Amylase and α-Glucosidase Inhibitory Activities Assay

Hyperglycemia-related metabolism can be effectively improved by inhibiting the activities of α-glucosidase and α-amylase. The results showed that the polysaccharides from many fungi could inhibit α-amylase and α-glucosidase to some extent (38). Therefore, the UAE *in-vitro* inhibitory effects of polysaccharides from *M importuna* against target enzymes for type 2 diabetes were investigated. As given in Figures 4D,E, MIP-D exhibited obvious inhibitory activities on α-amylase and α-glucosidase and their inhibitory activities were positively correlated with their concentration. At 2.0 mg/ml, the highest inhibitions of α-amylase and α-glucosidase in MIP-D were determined to be approximately 48.78 and 55.47%, respectively, but significantly lower than those of acarbose (69.85 and 93.87%). The IC_50_ value of α-glucosidase inhibitory activity of MIP-D was 0.15 mg/ml. Therefore, the results indicated that MIP-D exhibited excellent hypoglycemic activities and its inhibitory effect on α-amylase was stronger than that of α- glucosidase.

In summary, the results indicated that MIP-D presented excellent inhibitory effect on α-glucosidase and α-amylase, which could be explored further as a promising candidate for α-glucosidase and α-amylase inhibitors.

## Conclusion

In this study, ChCl-OA was selected from a series of solvents for the extraction of polysaccharides from *M. importuna*. Based on the results of single-factor experiment, BBD and RSM were utilized to identify the main parameters and optimize the extraction conditions. Next, the effects of two extraction techniques (UAE and HWE) on the yields and characteristics of polysaccharides (MIP-D and MIP-W) were evaluated. Under the most suitable conditions, the extraction yield of MIP-D was 4.5 times higher than hot water extraction. Moreover, MIPs had the similar functional groups, but their chemical compositions, monosaccharide contents, and molecular weights were different. The results of bioactivities indicated that MIP-D exhibited excellent antioxidant activity and inhibited α-amylase and α-glucosidase inhibitory activities. However, more experiments need to be performed in the future, such as purification and bioactivity *in vivo*. Besides, we need to determine the configuration of glycosidic bonds in MIP-D structure in the future study. Overall, this study not only provides a green, efficiency, and economical alternative method for the extraction of polysaccharides from *M. importuna*, but also a scientific basis for the comprehensive utilization of *M. importuna* as a potential functional food.

## Data Availability Statement

The original contributions presented in the study are included in the article/Supplementary Material, further inquiries can be directed to the corresponding author/s.

## Author Contributions

LX: conceptualization, methodology, investigation, supervision, and writing-review, and editing. XP: investigation and writing-original draft. YC: conceptualization and methodology. XG: software, formal analysis, and visualization. MC: resources, validation, and data curation. JM: resources and formal analysis. DG and RL: software and visualization. All authors contributed to the article and approved the submitted version.

## Conflict of Interest

The authors declare that the research was conducted in the absence of any commercial or financial relationships that could be construed as a potential conflict of interest.

## Publisher’s Note

All claims expressed in this article are solely those of the authors and do not necessarily represent those of their affiliated organizations, or those of the publisher, the editors and the reviewers. Any product that may be evaluated in this article, or claim that may be made by its manufacturer, is not guaranteed or endorsed by the publisher.
