# Supplementary material for: Ultrasound-Assisted Deep Eutectic Solvents Extraction of Polysaccharides From Morchella importuna: Optimization, Physicochemical Properties, and Bioactivities
Source: Front Nutr. 2022 Jun 9;9:912014. doi: 10.3389/fnut.2022.912014 (PMC9218490; doi:10.3389/fnut.2022.912014)
Supplement: Supplementary file 1 [file Data_Sheet_1.docx]

**Table S1.**

The synthesis of DESs by using choline chloride as the hydrogen bond acceptor and different hydrogen bond donors.

| Abbreviation | HBA | HBD | Molar ratio |
| --- | --- | --- | --- |
| DESs-1 | Choline chloride | Oxalic acid | 2:1 |
| DESs-2 | Choline chloride | Citric acid monohydrate | 2:1 |
| DESs-3 | Choline chloride | Acetamide | 1:2 |
| DESs-4 | Choline chloride | Urea | 1:2 |
| DESs-5 | Choline chloride | Glycerol | 1:4 |
| DESs-6 | Choline chloride | 1,4-butanediol | 1:4 |

HBA, hydrogen bond acceptor; HBD, hydrogen bond donor

**Table S2.**

Levels and code of extraction variables used in Box-Behnken design.

| Variable | Symbols | Coded levels | | |
| --- | --- | --- | --- | --- |
|  |  | -1 | 0 | 1 |
| Extraction time(min) | A | 20 | 30 | 40 |
| Extraction temperature (℃) | B | 50 | 60 | 70 |
| Liquid-solid ratio (v/w) | C | 1:20 | 1:30 | 1:40 |

**Table S3**

Box-Behnken experimental design and the results for extraction yield of MIPs.

| NO. | A | B | C | Extraction rate/% |
| --- | --- | --- | --- | --- |
| 1 | 1 | 0 | -1 | 4.96 |
| 2 | 0 | 1 | 1 | 5.18 |
| 3 | 0 | 0 | 0 | 5.93 |
| 4 | -1 | 0 | -1 | 4.75 |
| 5 | -1 | -1 | 0 | 3.57 |
| 6 | 0 | 0 | 0 | 5.94 |
| 7 | 0 | -1 | 1 | 4.74 |
| 8 | 0 | 0 | 0 | 5.78 |
| 9 | 1 | -1 | 0 | 4.56 |
| 10 | 0 | 0 | 0 | 5.83 |
| 11 | 1 | 0 | 1 | 5.29 |
| 12 | 1 | 1 | 0 | 4.93 |
| 13 | 0 | 1 | -1 | 5.14 |
| 14 | -1 | 1 | 0 | 4.87 |
| 15 | 0 | 0 | 0 | 5.74 |
| 16 | -1 | 0 | 1 | 5.04 |
| 17 | 0 | -1 | -1 | 4.31 |

**Table S4**

The IC_50_ values of DPPH, ABTS, OH radical scavenging activities and α-amylase and α-glucosidase inhibitory activities of MIP-D.

|  | MIP-D | Positive |
| --- | --- | --- |
| DPPH | 0.55±0.03 | 0.09±0.006 |
| ABTS | 0.75±0.05 | 0.21±0.01 |
| OH | 0.15±0.01 | 0.14±0.02 |
| α-amylase | NA | 1.03±0.06 |
| α-glucosidase | 0.15±0.01 | 0.07±.0.06 |

NA indicates that the data is not available.

**Fig.S1** UV spectrum of MIPs.
